# Preparation and Characterization of Modified Kaolin by a Mechanochemical Method

**DOI:** 10.3390/ma16083099

**Published:** 2023-04-14

**Authors:** Xiulin Liang, Qiang Li, Ying Fang

**Affiliations:** College of Materials Science and Engineering, Nanjing Tech University, Nanjing 211816, China

**Keywords:** kaolin, KH-570, mechanochemical method, characterization

## Abstract

A mechanochemical approach was utilized to prepare modified kaolin, and the hydrophobic modification of kaolin was realized. The study aims to investigate the changes in particle size, specific surface area, dispersion ability, and adsorption performance of kaolin. The structure of kaolin was analyzed using infrared spectroscopy, scanning electron microscopy, and X-ray diffraction, and the alterations to the kaolin microstructure were thoroughly researched and discussed. The results demonstrated that this modification method can effectively improve the dispersion and adsorption capacities of kaolin. Mechanochemical modification can increase the specific surface area of kaolin particles, reduce their particle size, and improve their agglomeration behavior. The layered structure of the kaolin was partially destroyed, the degree of order was debased, and the activity of its particles was enhanced. Furthermore, organic compounds were adsorbed on the surface of the particles. The appearance of new infrared peaks in the modified kaolin’s infrared spectrum suggested that the kaolin has undergone a chemical modification process, introducing new functional groups.

## 1. Introduction

The natural clay mineral kaolin has been used for many years [1]. It is a porous substance primarily made up of kaolinite and is a typical silicate mineral, with a crystal-chemical formula of 2SiO_2_·Al_2_O_3_·2H_2_O [2,3]. The ratio of silicon–oxygen tetrahedrons to aluminum–oxygen octahedrons is 1:1 and the two are linked by shared oxygen atoms. In the unit layered structure, oxygen atoms are connected around the silicon–oxygen tetrahedron, hydroxyl groups are connected around the aluminum–oxygen octahedron, and hydrogen bonds are formed by oxygen atoms, with hydroxyl groups connected between layers [4]. Kaolin is a cheap and abundant layered silicate mineral. However, unprocessed kaolin underperforms due to several structural, physical, and chemical limits, such as low purity, large particle size, hardness, and poor performance, which do not meet modern industrial production needs [5,6]. It has been confirmed that the specific surface area, catalytic activity, and adsorption performance of kaolin can be improved to a certain extent after modification by various methods. It has good reactive activity and adsorption capacities thanks to its unique layered structure, high specific surface area, and ion exchange capacity [7,8,9]. Therefore, a variety of modification methods are used to modify kaolin in order to maximize the value of kaolin in various industries and improve the economic efficiency of the industry. It is important to realize the comprehensive and reorganized utilization of kaolin resources [10,11].

According to the literature, research on the preparation of functional kaolin has received a lot of attention, both at home and abroad. In recent years, the main methods of kaolin modification have been investigated, including calcination modification, acid-base modification, coating modification, and organic modification [12,13,14,15]. Calcination modification can transform kaolin into amorphous metakaolinite, with high surface reactivity. Zhang et al. [16] indicated that unhydrated APTES could modify calcined kaolin under mechanical action at 80 °C, a modification which can significantly improve the hydrophobicity of the calcined kaolin. The optimum process conditions for kaolin calcination are affected by many factors, among which the most important are calcination temperature and calcination time [17,18,19,20]. Kaolin is calcined so that Al_2_O_3_, which was not originally reactive in the sample, becomes reactive. Its reaction with an acid or alkali produces substances that dissolve out of solution, and pores are formed on the surface of the particles of kaolin where Al_2_O_3_ was previously present, increasing its specific surface area and surface activity [13,21]. Powder agglomeration is effectively inhibited by coating modification. In addition to increasing the stability of the kaolin structure, the coating modification also optimizes the dispersion property and fluidity of the powder. The reaction conditions and the size of the specific surface area of the kaolin particles are the major factors regulating the efficacy of a surface coating [22]. The mechanism behind organic modification is to apply a substance to the surface of kaolin particles, effectively inhibit the agglomeration of the powder, and reduce their surface energy [23]. Organic modification can address the drawbacks of materials susceptible to embrittlement, increase compatibility between kaolin fillers and polymeric materials, and perform better [24,25]. Ahmed K. Sakr et al. [26] considered that clay is a good material for absorbing uranium and has a high adsorption capacity. The results showed that the adsorption capacity of the adsorbent for uranium was 175.10 mg/g at pH 4.5, with an adsorption time of 60 min at 25 °C. Modified kaolin with high activity and adsorption capacity can be prepared by various activation or surface modification methods, and can be used to treat various pollutants in water with excellent adsorption effects [27,28,29].

In the last few years, the mechanical–chemical method of modification of mineral powders has become a research topic in which the modification method combines ultrafine grinding and surface modification [30]. The modifier is added to the grinding process, and the internal energy of the system is increased by using the mechanical force in the grinding process so that the specific surface area of the material is increased, and the lattice defects are increased. The modifier interacts with the powder and covers the surface of the mineral micro-powder to prepare the ultrafine-modified kaolin [31,32]. The surface modifier mainly uses various functional chemical agents, such as silane coupling agents and organic acids. The surface properties of kaolin particles are effectively regulated by mechanochemical modification on the surface of the particles; therefore, the purpose of the modification is achieved [33,34].

Although there have been many studies on the modification of kaolin, there has been little work on the modification of kaolin by combining mechanical ball milling and surface chemical modification. This modification method has the advantages of being a simple process, with low economic cost, being green (pollution-free), and demonstrating good modification effect. Therefore, in this article, the organic modification of kaolin by direct ball milling uses mechanical force and chemical effects in order to study the pattern of change in kaolin, in terms of particle size, specific surface area, dispersion properties, and adsorption properties. In order to study the microstructure of kaolin materials, characterization was performed through X-ray diffraction, scanning electron microscopy, and infrared spectroscopy.

## 2. Experimental Procedure

### 2.1. Materials

For this study, kaolin was purchased from Shanghai McLean Biochemical Technology Co., Ltd. (Shanghai, China), with a mesh of 800, D_50_ of 4.22 μm, and a specific surface area of 24,100 cm^2^/g. Its chemical constituents are listed in Table 1. The modifier used was KH-570 (BR, 98%), purchased from Shanghai Yuan Ye Biotechnology Co., Ltd. (Shanghai, China). Its molecular formula is C_10_H_20_O_5_Si. Methyl orange (AR) was purchased from Tianjin Chemical Reagent Research Co., Ltd. (Tianjin, China) Liquid paraffin (CP) was purchased from Shanghai Lingfeng Chemical Reagent Co., Ltd. (Shanghai, China). Deionized water was used throughout the study.

### 2.2. Preparation of Sample

Firstly, 15 g of the original sample was weighed and dried at 105 °C, and then ground in a planetary ball mill. The pre-hydrolyzed modifier (KH-570) was added during mechanical milling. The following ball milling parameters were used: the main disc speed was set to 300 r/min, the powder-to-ball ratio was set to 1:5, the ratio of the small ball to the big ball was set at 3:1 (the ball sizes were 8 mm and 10 mm, respectively), and the ball milling medium was agate ball. The ball milling times were 1, 2, 3, 4, 5, and 6 h, with the amount of modifier being 0%, 1%, 2%, and 3% of kaolin mass. Finally, the modified samples were dried and stored in a desiccator. Each sample was named Kxy, where x is the ball milling time and y is the amount of modifier added. For example, K12 represents the original sample ball milling time of 1 h where the modifier addition amount is 2%.

### 2.3. Dispersivity Experiments

A 1.0 g kaolin sample was added to 10 mL liquid paraffin. The kaolin sample was fully dispersed in the solvent for 30 min by magnetic stirring, and the settling of kaolin samples in liquid paraffin for 10, 20, 30, 40, 50, and 60 min was recorded. Each experiment was repeated three times to calculate the average.

### 2.4. Adsorption Experiments

To study the change of adsorption properties of kaolin before and after modification, 0.5 g kaolin was added to 100 mL methyl orange solution with a concentration of 10 mg/L, the pH of the solution remained unchanged, and magnetic stirring was performed for 30 min. After standing, the supernatant was centrifuged for 10 min. The absorbance was measured at 465 nm by a visible spectrophotometer. Each experiment was repeated 3 times and the average was calculated. The removal rate *R* (%) was calculated by the following formula:

(1) $$R=\frac{C_{0}-C_{e}}{C_{0}}\times 100\%$$

where *C*_0_ (mg/L) is the initial concentration of the solution and *C_e_* (mg/L) is the equilibrium concentration of the solution.

### 2.5. Characterization of Samples

The XRD pattern was tested by an X-ray diffraction meter (XRD) (SmartLab TM, Rigaku, Akishima, Japan) with a Cu target. The power was 3 kW, the XRD scanning range was 10–80°, the step size was 0.02°, and the scanning speed was 10°/min.

The effect of mechanical ball milling on the particle size of kaolin was analyzed by a laser particle size analyzer (Mastersizer 2000, Malvern, PA, USA). The laser particle size analyzer also calculates the specific surface area of the particles while measuring their size.

The microstructure of the samples was observed by scanning electron microscopy (SEM) (Regulus 8100, Hitachi, Japan), and the accelerating voltage was 5 kV.

Fourier transform infrared spectroscopy (FTIR) (Nicolet iS20, Thermo Scientific, USA) was used in order to study the changes in the surface functional groups of kaolin before and after modification. The wave number range was 400–4000 cm^−1^.

## 3. Results and Discussion

### 3.1. Changes in Particle Size

**Figure 1:**
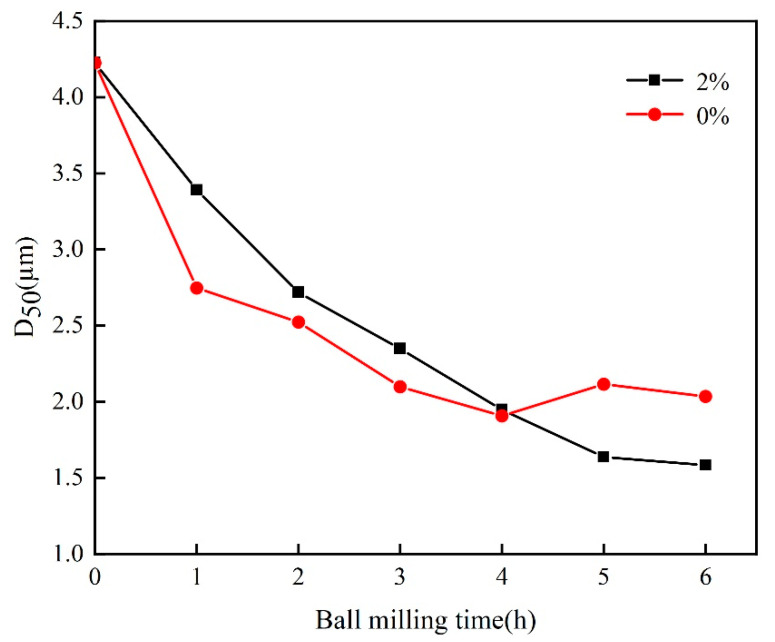
The change of D_50_ of kaolin at different milling times when adding a modifier.

Figure 1 shows the trend of the average particle size D_50_ of kaolin at different ball times when the modifier is added. The results indicated that the average particle size of kaolin without a modifier decreased significantly at the initial stage of the ball milling, while, with the extension of the ball milling time, the curve of the average particle size decline of kaolin became more gradual. After 4 h of ball milling, the average particle size did not continue to decrease but rather started to increase. This is due to the agglomeration of particles under the action of van der Waals forces and electrostatic forces, both of which increase the size of particles.

When the content of the modifier is 2%, with the increase in ball milling time, the average particle size of the particles gradually decreased, which indicates that the modifier was combined with the kaolin particles to reduce the surface energy, inhibit powder agglomeration behavior, and effectively reduce the particle size of the kaolin [35,36]. After ball milling for 6 h, the average particle size of the modified kaolin reached 1.58 μm.

### 3.2. Particle Size Distribution

**Figure 2:**
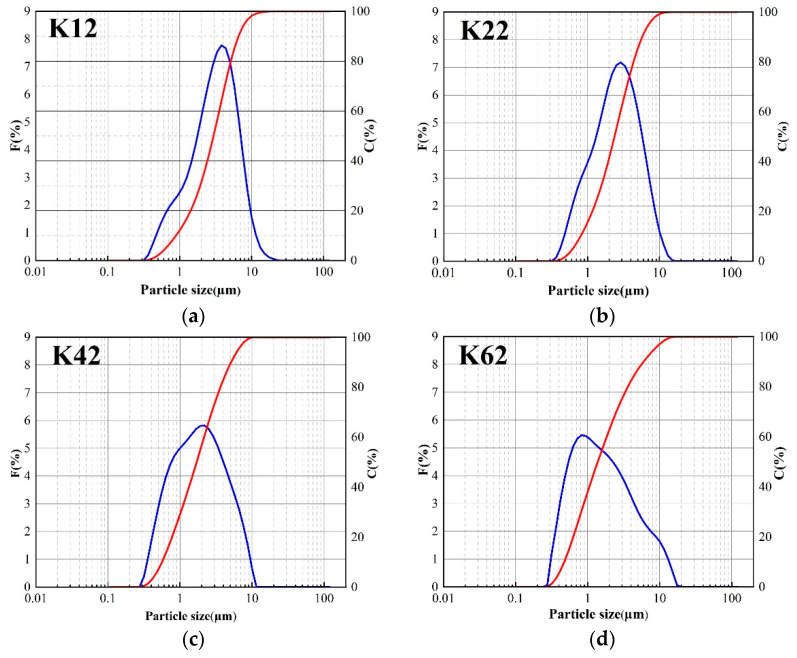
Particle size distribution at different ball times (**a**–**d**).

Figure 2 shows the particle size distribution of kaolin modified for 1 h, 2 h, 4 h, and 6 h. The left ordinate represents the frequency distribution (F%), represented by a blue line, and the right ordinate represents the cumulative distribution (C%), represented by a red line. It is discovered that the particle size of kaolin gradually decreases with an increase in ball milling time, as can be observed in diagrams (a) to (d). This is because the particle size of kaolin gradually decreases under mechanical grinding, leading to changes in the particle size distribution as the ball milling time increases. Particles with a cumulative distribution of approximately 2 µm in size become more common with increasing milling time. After milling the ball for 6 h, the cumulative distribution of particles with a 2 µm particle size can reach 61.3%.

### 3.3. Changes in Specific Surface Area

The specific surface area of powder materials can be obtained by theoretical calculation of actual particle size distribution data [37]. The particle size analyzer calculates the specific surface area of the particles while measuring the particle size, and the calculation formula is shown in Equation (2):

(2) $$Sw=\varphi_{sv}\rho^{-1}\int_{0}^{\infty }\frac{F^{\prime }\left(D\right)}{D}dD$$

where *φ_sv_* is the specific surface area shape coefficient, *ρ* is the density of particles, *F*′(*D*) is the frequency function of particle size distribution, and *F*′(*D*) *Dd* is the percentage of particle volume between *D* and *D + dD*.

**Figure 3:**
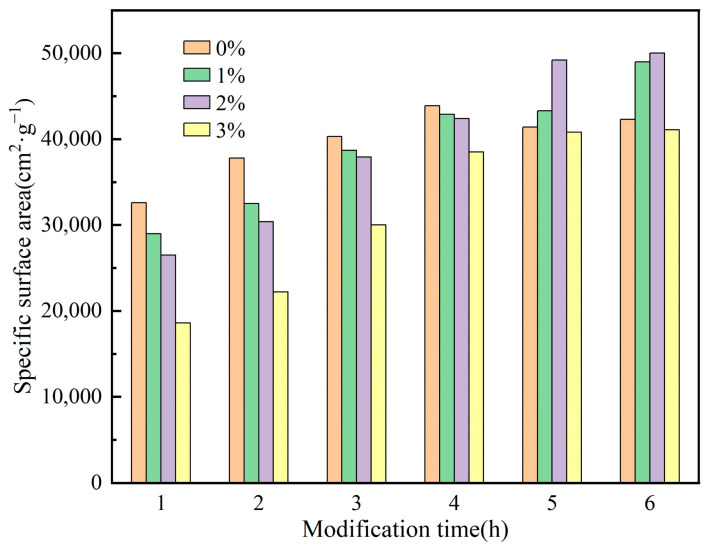
Variation of the specific surface area of kaolin at different ball milling times.

Figure 3 shows that the specific surface area of kaolin initially increases and then decreases when the modifier content is 0%, due to the agglomeration behavior of particles during ball milling. This pattern is consistent with the change curve of particle D_50_. As the milling time increases, the specific surface area of kaolin with different amounts of the modifier gradually increases. It can be inferred that the modifier improves the agglomeration behavior of particles, enlarges the specific surface area, increases surface energy, and enhances kaolin adsorption [38]. By comparing the specific surface areas in Figure 3, it can be concluded that adding a 2% modifier is the most effective. After 6 h of ball milling with a 2% modifier, the specific surface area of kaolin can reach 50,000 cm^2^/g. Note that in the early stage of ball milling, the specific surface area of particles decreases with an increase in the modifier content. This may be because the modifier primarily acts as a wetting agent, reducing the friction between particles, which is not conducive to mechanical ball milling.

### 3.4. Dispersion Ability Test

**Figure 4:**
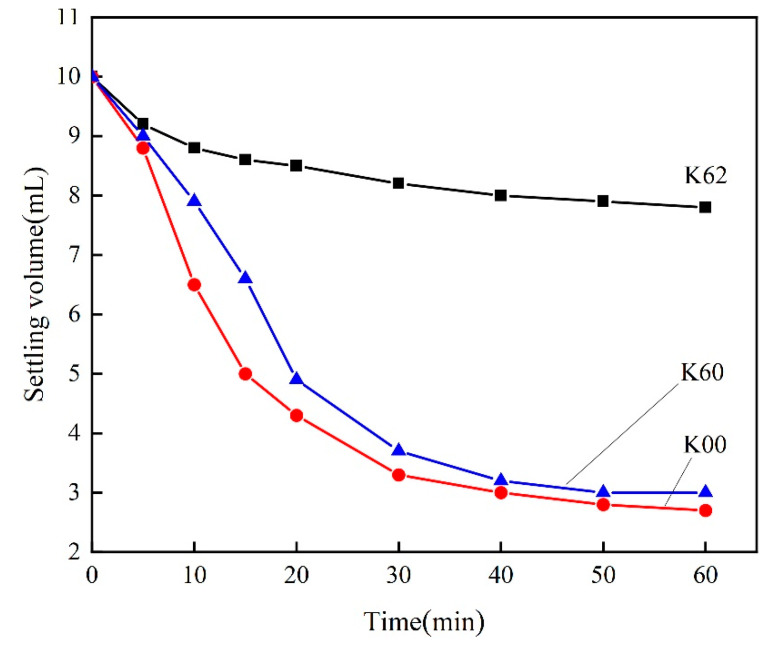
Variation of the sedimentation volume of kaolin in liquid paraffin.

Figure 4 illustrates the sedimentation volume change curve of kaolin in liquid paraffin before and after modification. The sedimentation behavior of kaolin in solvents can reflect its compatibility with the solvent; the larger the sedimentation volume in the solvent, the slower the sedimentation rate, and the better the dispersion in the solvent [39,40]. As shown in Figure 4, the settlement curves of the original sample (K00) and direct ball milling (K60) are steep at the initial stage of settlement, with a fast-settling velocity. In contrast, the modified kaolin (K62) exhibits a slow sedimentation rate in paraffin and a large sedimentation volume. This indicates that the mechanical and chemical composite modification improves the hydrophobicity of the modified kaolin, and enhances its compatibility with organic solvents.

### 3.5. Adsorption Performance Test

**Figure 5:**
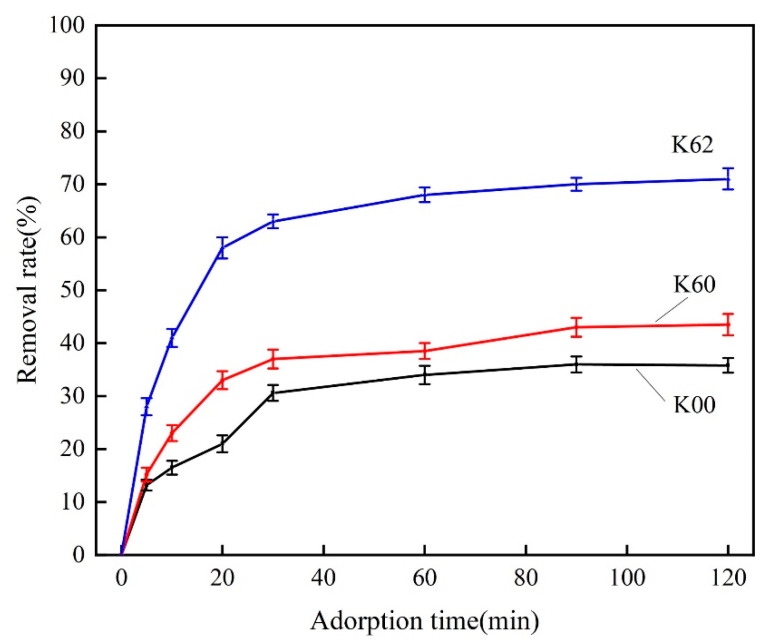
Adsorption of methyl orange solution on kaolin before and after modification.

Figure 5 shows the adsorption curves of samples K00, K60, and K62 for methyl orange solution. In order to investigate the change in the adsorption performance of kaolin before and after modification, the removal rate of kaolin for methyl orange solution was determined by spectrophotometry. As shown in Figure 5, the adsorption curves of the original sample (K00) and direct ball-milled kaolin (K60) are relatively flat, and the removal rate increases slowly with the increase in adsorption time. However, the removal rate of modified kaolin (K62) from methyl orange solution is significantly higher than that of the original sample K00 and sample K60. There are two possible reasons for this improvement. Firstly, the reduction in the size of kaolin particles leads to an increase in specific surface area, thereby enhancing its adsorption performance. Secondly, the coating of KH-570 on the kaolin surface generates new functional groups, thereby improving its lipophilicity. As a result, mechanochemical modification can alter kaolin’s adsorption performance and enhance its ability to adsorb organic matter.

### 3.6. Microstructure

#### 3.6.1. XRD Analysis

**Figure 6:**
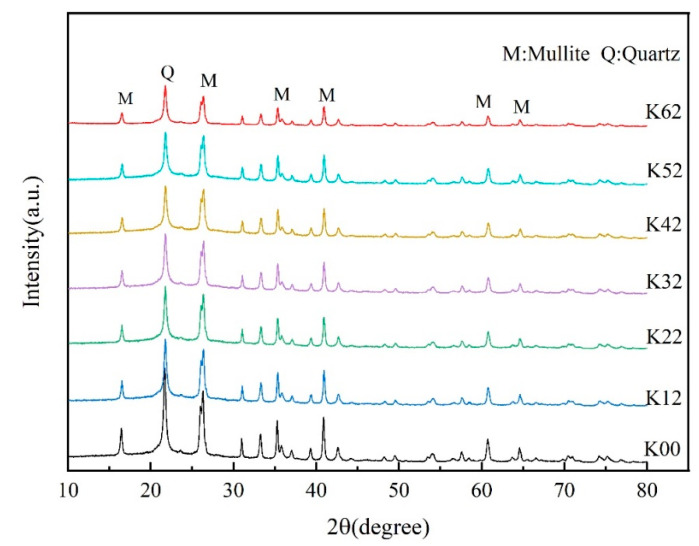
XRD diffractograms of original and modified kaolin.

Figure 6 shows the XRD diffraction pattern of kaolin before and after modification. As shown in the figure, the original sample (K00) has characteristic peaks of kaolin structure at 16.48°, 21.76°, 26.32°, 35.3°, and 41.1°. Moreover, the diffraction peaks are narrow, the peaks are symmetrical, the crystal form is good, and there is a “mountain”-shaped diffraction peak between 35° and 45°, which is very obvious and has high crystallinity. Compared to the original kaolin, the modified kaolin shows a certain degree of reduction in the intensity of the main diffraction peaks, indicating that the mechanochemical modification decreased the order of kaolin to some extent. However, the shape of the diffraction peaks remained unchanged, indicating that the main structure of kaolin remained the same. After mechanochemical modification, the main diffraction peak position did not move greatly, indicating that the modification did not transform the interlayer spacing of kaolin, but only modified the surface of kaolin; the modifier, KH-570, did not enter the interlayer of kaolin. With an extension in ball milling time, the weak binding force between the layers causes partial destruction of the layered structure of kaolin, while the internal structure remained unchanged. The interlayer water and structural water were removed, resulting in enhanced amorphous crystal formation. The greater the degree of amorphousness of the crystals, the stronger the activity of the particles, which requires a longer grinding time for the powder [41,42].

#### 3.6.2. SEM Analysis

**Figure 7:**
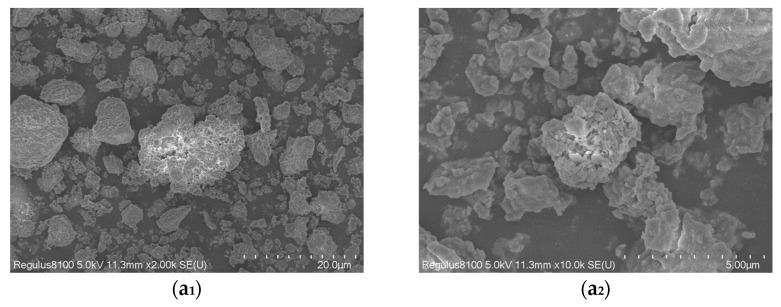

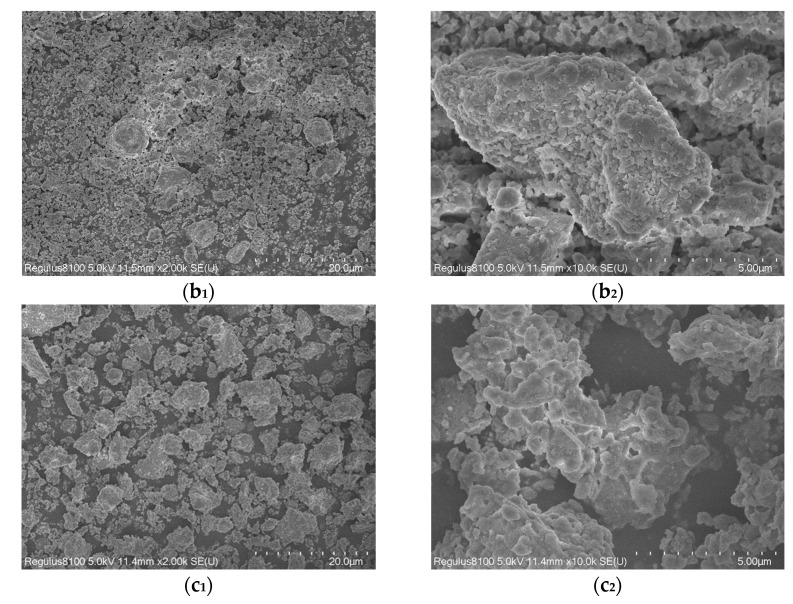
SEM image of kaolin (**a_1_**,**a_2_**) K00, (**b_1_**,**b_2_**) K60, and (**c_1_**,**c_2_**) K62, at 2K× and 10K×, respectively.

SEM images of samples K00, K60, and K62 are presented in Figure 7, at magnifications of 2000× and 10,000×. As shown in Figure 7(a_1_,a_2_), the original sample is mainly kaolinite, with a flaky shape, dense structure, and rough surface. Additionally, small aggregates can be observed on the surface of sample K60 in Figure 7(b_1_,b_2_). This is due to the grinding of large particles into small particles, which are then agglomerated together. Figure 7(c_1_,c_2_) show that organic compounds have been adsorbed on the sample surface. Compared with the original sample, the particle size of the sample was significantly reduced, and the particles were in a fragmented state, which was mainly caused by the mechanical milling effect. These structural characteristics not only increase specific surface area, but also improve porosity ratio and pore smoothness. Therefore, the kaolin with this structure exhibits better adsorption performance. It can be inferred that the mechanochemical method significantly improved the surface properties of kaolin, which led to partial irregularity in the shape of the particles and reduced crystal orderliness on the surface of kaolin.

#### 3.6.3. FT-IR Analysis

**Figure 8:**
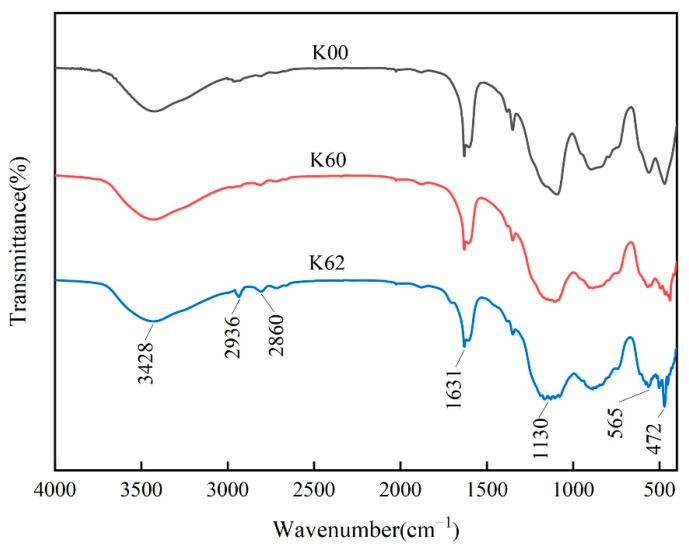
FT-IR spectra of kaolin before and after modification.

Figure 8 shows the infrared spectra of samples K00, K60, and K62. It can be observed from the figure that the infrared spectra of the sample without modifier after ball milling for 6 h (K60) and the original sample (K00) are basically the same, and the infrared spectrum of the K60 sample does not have the characteristic peak of KH-570. In contrast, kaolin (K62) milled with modifiers has two new infrared peaks, CH_3_ and CH_2_, located at 2936 cm^−1^ and 2860 cm^−1^, respectively. The appearance of new infrared peaks indicates that KH-570 has been coated on the surface of kaolin and new functional groups have been generated [43]. In addition, the characteristic peak of 3428 cm^−1^ of kaolin did not change significantly after ball milling, indicating that the layered structure of kaolin was partially destroyed, but the internal structure remained largely unchanged. The characterization results are also consistent with the XRD analysis results. The OH group vibration adsorption peak of H-O-H in kaolin was observed at 1631 cm^−1^, and the stretching vibration adsorption peak of Si-O was at 1130 cm^−1^. The adsorption peak of K62 at 565 cm^−1^ was attributed to the bending vibration of Al-O. The bands around 472 cm^−1^ were sharp and strongly overlapping, almost merging into one band. The enhancement of the peak indicates that more Si-O-Si was generated during the modification process.

In this study, we found that the adsorption peak of OH vibration was significantly weakened after modification by the mechanochemical method, which may be caused by the chemical reaction between the silanol formed by the hydrolysis of the hydrophilic group of KH-570 and the hydroxyl group on the surface of kaolin [44]. This may also be related to the effect of mechanical ball milling, which to a certain extent destroys the layered structure of kaolin crystals; breaks the crystal valence bonds, such as Si-O bonds and Al-O bonds; produces a large number of silicon and aluminum dangling bonds; and makes it highly reactive. These dangling bonds can easily react with the silanol produced by the hydrolysis of the silane coupling agent, resulting in a condensation reaction [45].

## 4. Conclusions

In this work, a mechanochemical approach was utilized in order to modify the surface of kaolin. Kaolin was prepared by controlling the milling time and the amount of modifier added. The dispersion, adsorption, specific surface area, particle size distribution, and microstructure of kaolin were analyzed and discussed. The specific conclusions are as follows:

Through mechanochemical modification, the hydrophobicity of the powder was improved to some extent. A layer of organic matter was adsorbed onto the particle surface, thereby improving the adsorption performance of kaolin. With the addition of the modifier, the agglomeration behavior of the particles improved. At the end of the 6 h ball milling period, the specific surface area reached 50,000 cm^2^/g, and the cumulative distribution of 2 µm particles reached 61.3%. Part of the layered structure of kaolin was destroyed after mechanochemical action, thus reducing the order degree of kaolin. This structural state increased the specific surface area and had obvious advantages in terms of adsorption performance. New infrared peaks appeared in the infrared spectrum of the modified kaolin, indicating that KH-570 had been combined with kaolin.

## Figures and Tables

**Table 1:** Chemical constituent of kaolin, measured in wt.%.

Composition	Al_2_O_3_	SiO_2_	P_2_O_5_	SO_3_	K_2_O	CaO	TiO_2_	Fe_2_O_3_	Others
Content(wt.%)	47.38	50.71	0.03	0.02	0.15	0.20	0.87	0.59	0.05

## Data Availability

Not applicable.
